# Supplementary material for: Genomic signatures of convergent shifts to plunge-diving behavior in birds
Source: Commun Biol. 2023 Oct 24;6:1011. doi: 10.1038/s42003-023-05359-z (PMC10598022; doi:10.1038/s42003-023-05359-z)
Supplement: Supplementary file 2 — Supplementary Information [file 42003_2023_5359_MOESM2_ESM.pdf]

Supporting Information for

Genomic signatures of convergent shifts to plunge-diving behavior in birds

Chad M. Eliason[1,2], Lauren E. Mellenthin[3], Taylor Hains[1,2,4], Jenna M. McCullough[5], Stacy Pirro[6], Michael J. Andersen[5], Shannon J. Hackett[2,4]

Corresponding:
Chad M. Eliason
Email: celiason@fieldmuseum.org

This PDF file includes:

Supplementary Notes 1–4
Supplementary Figures 1–13
Supplementary Tables 1–2
Supplementary references

Supplementary Note 1: Development and pattern genes are under positive selection across kingfishers

We looked for evidence of genes under positive selection across the kingfisher phylogeny (i.e., not just in plunge-diving lineages). After filtering incomplete and nonsense genes[1], we identified several genes putatively under positive selection in kingfishers (Fig. 3c). Genes under positive selection across kingfishers but not convergent within plunge-diving species (n = 389) include those related to pattern specification, embryonic morphogenesis, skeletal system development, and brain development (Supplementary Fig. 12). One gene of interest was *BMP4*, a known regulator of beak shape in birds[2]. Yet, analysis of the relationship between beak shape data[3] and rate of molecular evolution in the *BMP4* gene showed a non-significant slightly negative relationship (RERconverge test, $\rho = -0.004$, $p = 0.98$).

Supplementary Note 2: Taste receptor genes are under positive selection across kingfishers

In addition to our genome-wide scan, we also focused on a target set of eleven avian taste receptor genes, as their evolution has been suggested to be related to fish-eating behavior in penguins[4]. We found that eight (73%) of these taste receptor genes were positively selected in kingfishers. Interestingly, two genes that were not positively selected in kingfishers (*PKD2L1*, *SCNN1A*) were found to be under positive selection in a broader analysis across birds[5]. In particular, one gene shown to be related to fish-eating behavior in penguins, *TAS1R3*[4], was found to be under positive selection in kingfishers (Supplementary Data 3) but not across birds[5]. To further test whether there was a significant shift in positive selection regime for the *TAS1R3* gene in crown kingfishers relative to other birds, we merged our kingfisher alignment with an avian gene dataset[5] and compared two sets of models in PAML. We first tested whether a model allowing positive selection at some sites in kingfishers was a better fit to the data than a model (M0) in which selection is neutral across the genome. This comparison showed strong support for positive selection (Supplementary Table 2). We next fit a branch-site model to test whether crown kingfishers have stronger positive selection than other birds. We found that eight amino acid sites were under strong positive selection (Bayesian posterior > 0.95; Supplementary Table 2), but only in crown kingfishers. Most of these sites were outside the transmembrane region of the *TAS1R3* gene[6]. Interestingly, these positions differed from sites under positive selection in the hummingbird *TAS1R3* gene[6], as well as from sites we identified as convergently evolved in PCOC analyses (Supplementary Fig. 13), with the exception of amino acid 578 (Supplementary Table 2). Although we cannot conclude that these shifts result in functional changes in taste perception, they do either point to relaxed selection in plunge-diving lineages[7] or convergence in gene function despite differences in amino acid mutations. As an example of the latter, high-altitude birds show different mutations in hemoglobin genes, yet converge at the level of gene function[8]. Further functional genomics work could clarify the role of *TAS1R3* in kingfisher behavior and evolution[9].

Supplementary Note 3: Convergent rate shifts are concentrated in coding regions of the genome

To determine rapidly evolving genes at the whole sequence level, we used RERconverge to identify accelerations or decelerations in the rate of molecular evolution linked to changes in behavior. Given the more indirect/multidimensional relationship between behavior and morphology, we predicted that more regulatory and coding shifts would be associated with morphology compared to plunge-diving behavior. We found significant support (FDR < 0.15) for convergence in molecular rates of evolution in 21 genes associated with plunge-diving behavior (Supplementary Fig. 4b). Although we did not identify any significantly enriched GO terms, accelerated genes (n = 14) were involved in protein modification (Supplementary Fig. 4e), while decelerated genes (n = 7) were associated with liver regeneration (*GFER*) and T cell activation (*NFATC1*), among other functions. These numbers are comparable to a recent study in spiders showing a small number of genes with convergent rate shifts (n = 7), despite a large number of genes (n = 671) showing positive selection in at least one amino acid being linked to convergent behavior[10]. For regulatory regions, we identified 37 conserved noncoding elements (CNEs) significantly associated with plunge-diving behavior (Supplementary Fig. 4c). Accelerated CNEs (n = 26) were involved in anatomic structure morphogenesis and circulatory system development (Supplementary Fig. 4f). Decelerated CNEs (n = 11) were involved in cardiac function (e.g., *GATA5*). Overall, we found proportionally more rate-shifted genes than CNEs linked to convergent foraging behavior (odds ratio, OR = 2.06, p = 0.01). By contrast, recent work on morphological convergence has found support for shifts in noncoding regions of the genome and few protein-specific trends associated with these phenotypes[11,12].

Supplementary Note 4: Comparing methods for identifying signals of convergent positive selection.

The "drop test" branch-site (BS2) model produced the largest list of putative positively selected convergent genes in plunge-diving kingfishers (n = 512; Supplementary Fig. 5). The CBUST approach identified 337 genes, but showed the most overlap with our conservative set of 93 genes (Supplementary Fig. 5). Overlapping genes included *MAPT, CENPJ, and ATRIP* (see Supplementary Dataset 3 for complete list). An example of a gene identified as significant by CSUBST but not the hybrid approach was *COL27A1* (Supplementary Data 5), *a* gene that has been linked to beak length variation in songbirds[13]. The modified BS2 model overlapped in some genes (e.g., *BBS10*) but also showed novel target genes compared to the hybrid approach, including *BGLAP,* recently identified as being associated with rapid beak shape evolution across birds[14], and *TYRP1*, a gene involved in melanin pigmentation (see Supplementary Data 4 for complete list). These results highlight the importance of considering alternative approaches in identifying sets of positively selected genes at broad phylogenetic scales (see ref. [15] for discussion).

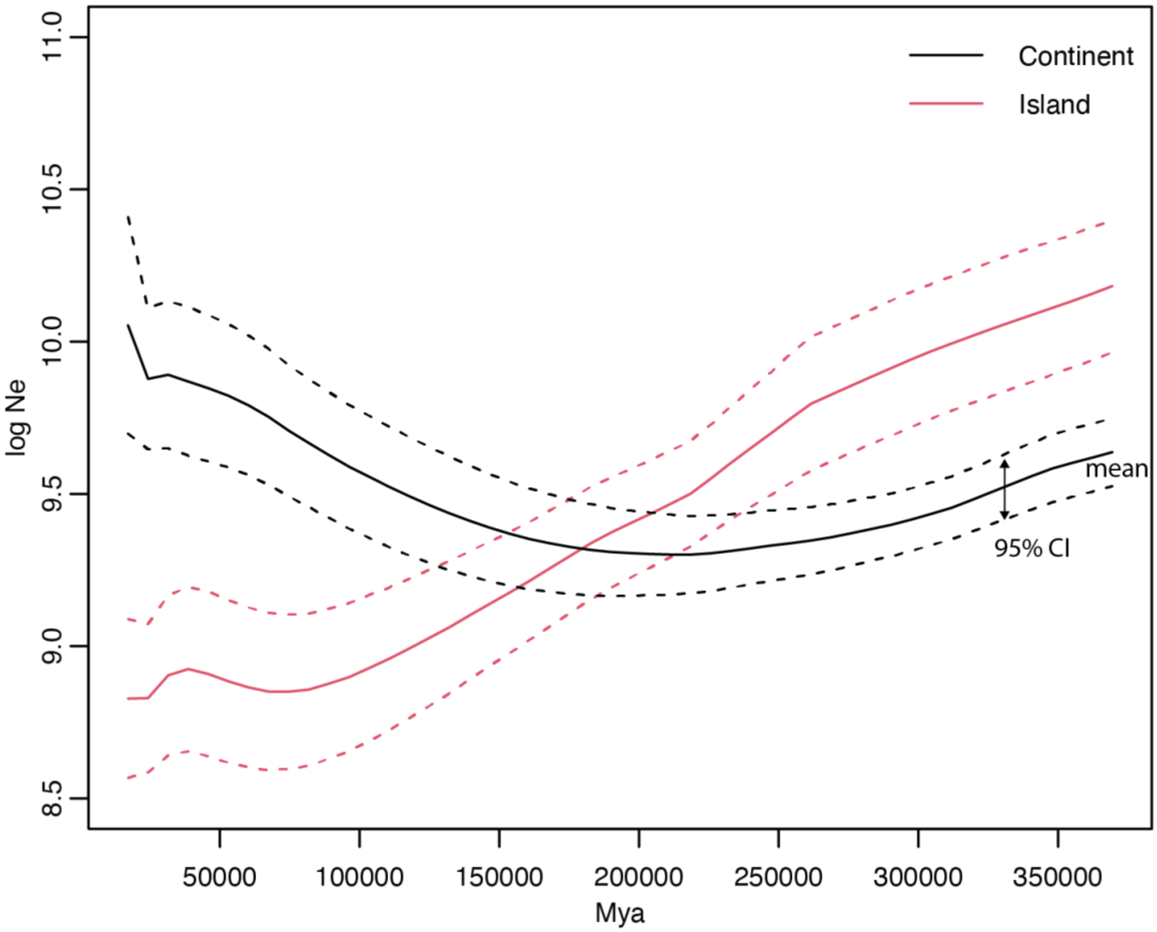

Supplementary Fig. 1. Effective population size through time. Results of PSMC analyses from n = 31 species' genomes. Lines show average values (solid lines; clipped to maintain temporal overlap for all species; see Fig. 1) and 95% confidence intervals (dashed lines) for islands (red) and continents (black). This relationship was significant ($p < 0.05$).

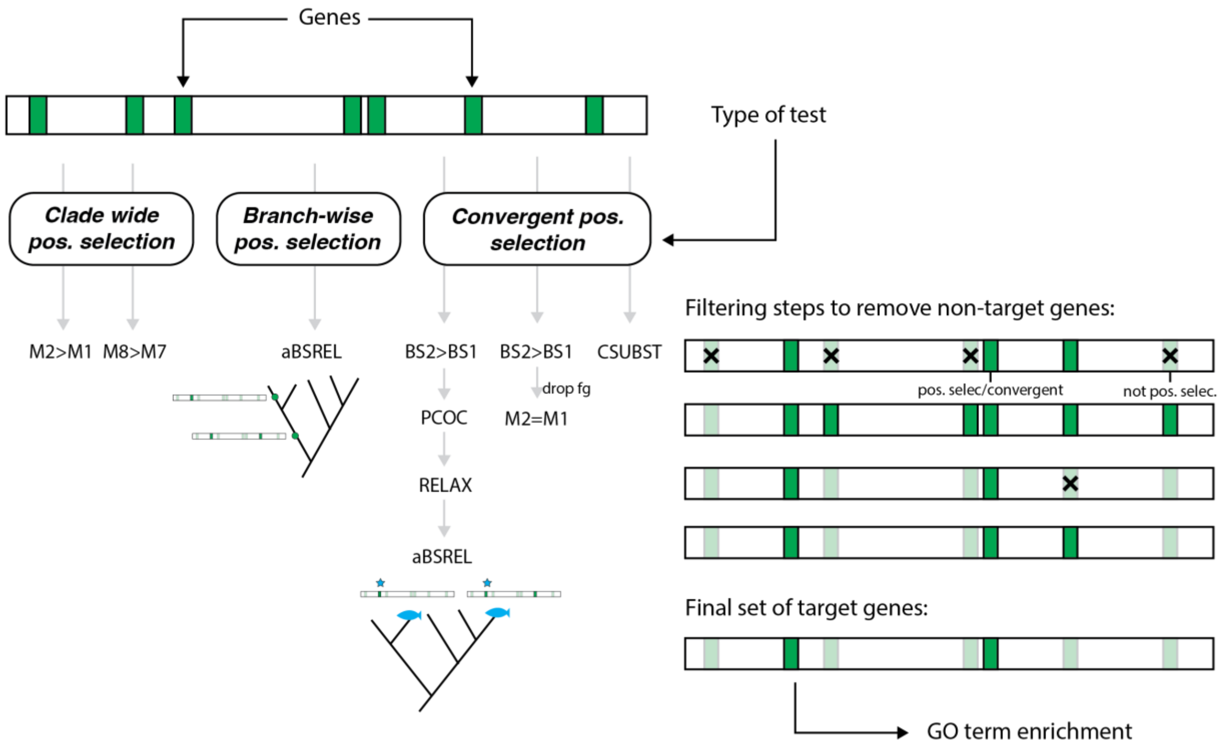

Supplementary Fig. 2. Flowchart for studying trends in molecular evolution across kingfishers. Genes depicted as green boxes. Genes were filtered in various ways to determine target sets of genes that were used in downstream enrichment analyses. The background gene set was determined from the genes annotated in the collared kingfisher genome.

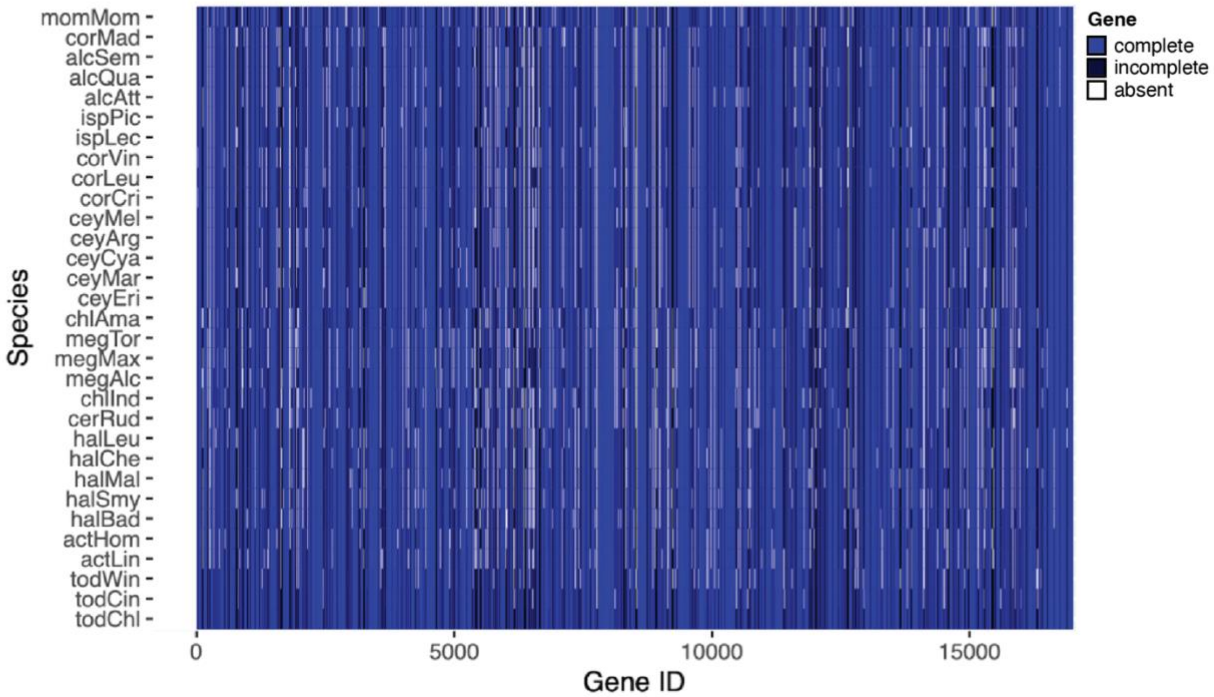

Supplementary Fig. 3. Heatmap showing gene space completeness relative to the reference genome. Rows indicate species and columns indicate genes annotated in the reference genome (*Todiramphus chloris*, todChl). Colors represent genes that are complete and present in all species (blue), incomplete in some species but present in all species (black), and genes that are missing in certain species (white). Species on the y-axis are sorted by phylogenetic distance (in millions of years) to the reference. The pattern suggests weak phylogenetic signal in the distribution of missing genes among species and increases confidence in our reference mapping approach.

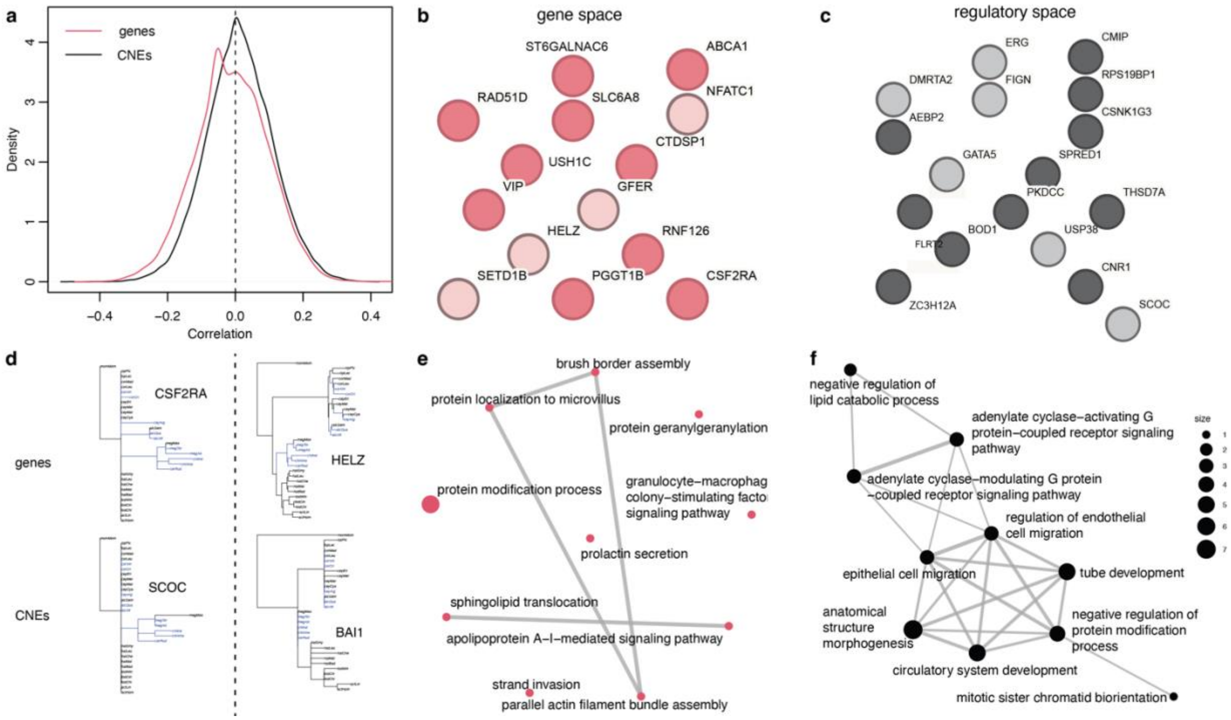

Supplementary Fig. 4. Genic and regulatory regions showing convergent rate shifts in plunge-diving lineages. a Density plot showing distribution of correlation coefficients (Spearman's ρ) between plunge-diving and rates of molecular evolution in genic (red) and regulatory regions (black). **b,c** Annotated genes showing evidence for significant change in rate of evolution for genic (**b**) and regulatory regions (**c**), determined using bedtools closest. Shading of circles indicates accelerated (darkly shaded) and decelerated (lightly shaded) genic or regulatory regions. **d** Exemplar trees for significant deceleration (left) and acceleration (right) in plunge-diving lineages (highlighted in blue). **e,f** GO term network plots for accelerated genic (**e**) and regulatory regions (**f**). Size of nodes indicates number of genes that are linked to that GO term. Note: there were no significantly enriched GO terms (i.e., all FDRs > 0.5).

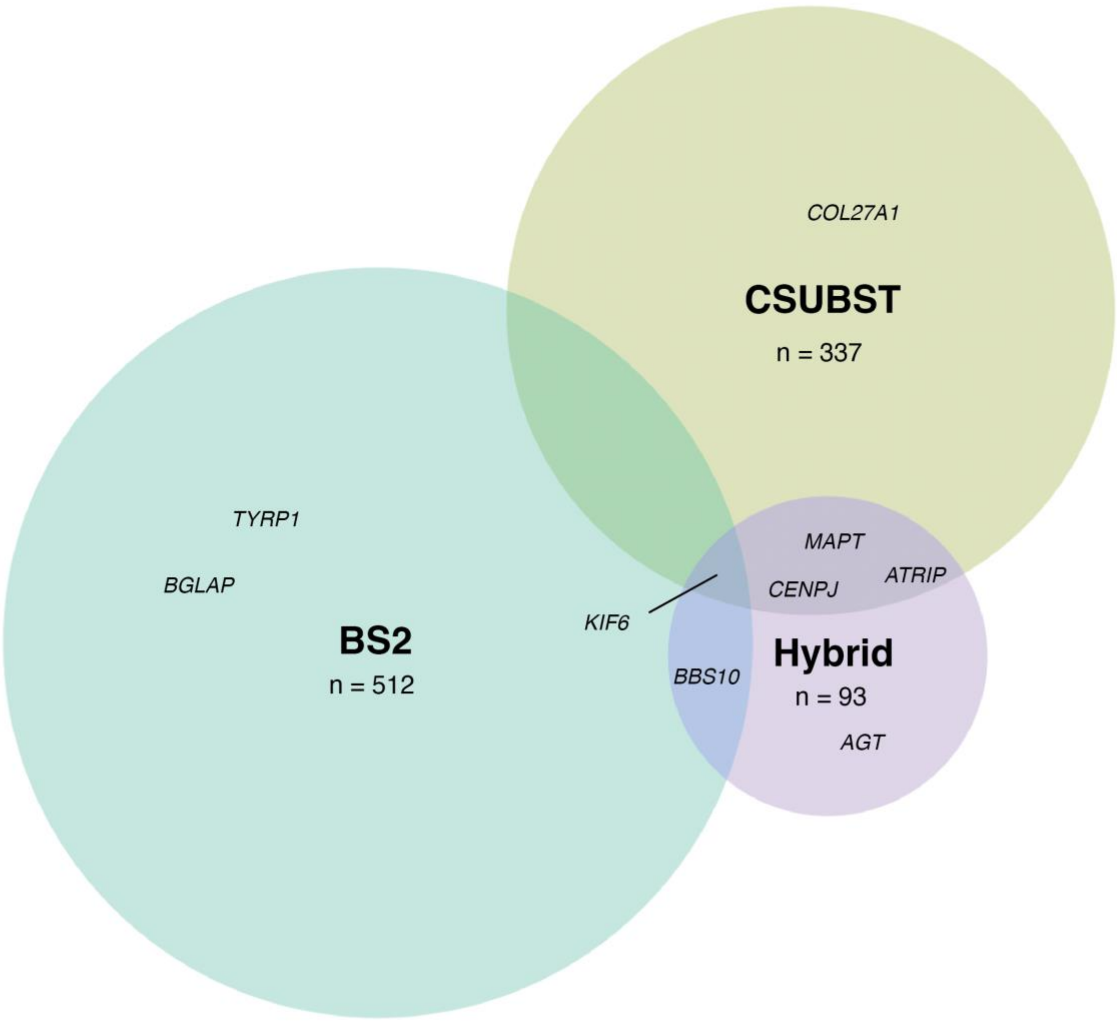

Supplementary Fig. 5. Comparison of different approaches for identifying convergent positively selected genes. Venn diagrams produced with the R package venneuler show overlap between different approaches of identifying sets of target genes. Examples of genes discussed in the text are listed. See Supplementary Methods for details.

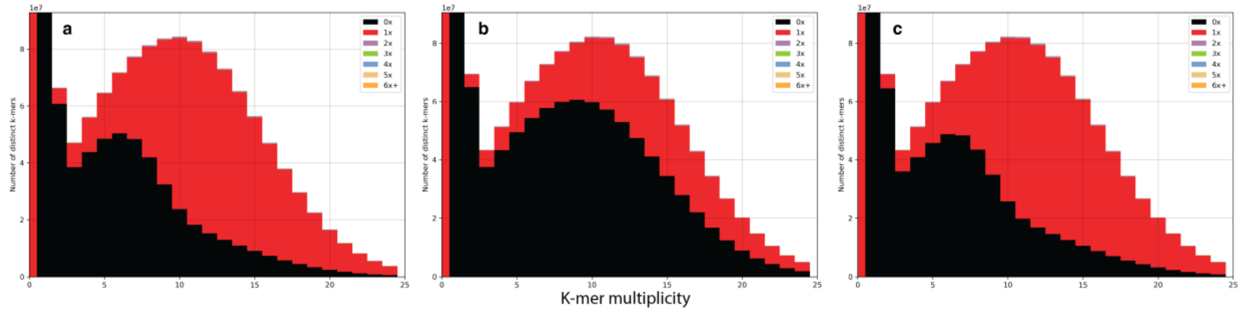

Supplementary Fig. 6. Assessing different read-mapping approaches for reference-guided genome assembly. a-c K-mer plots were produced using the K-mer Analysis Toolkit (KAT) program[16] by mapping reads to a pseudo-reference generated by calling variants using BWA-MEM (**a**), LAST (**b**), and NGM (**c**). Histograms show proportion of reads mapped 1X to the pseudo-reference (red) versus unmapped reads (black). An ideal distribution would have all reads mapped 1X. Note BWA-MEM is similar in performance to NGM known to be ideal for mapping reads to divergent species. Reads were sequenced from tissue for Hombron's kingfisher (*Actenoides hombroni*) based on FMNH specimen number 472665.

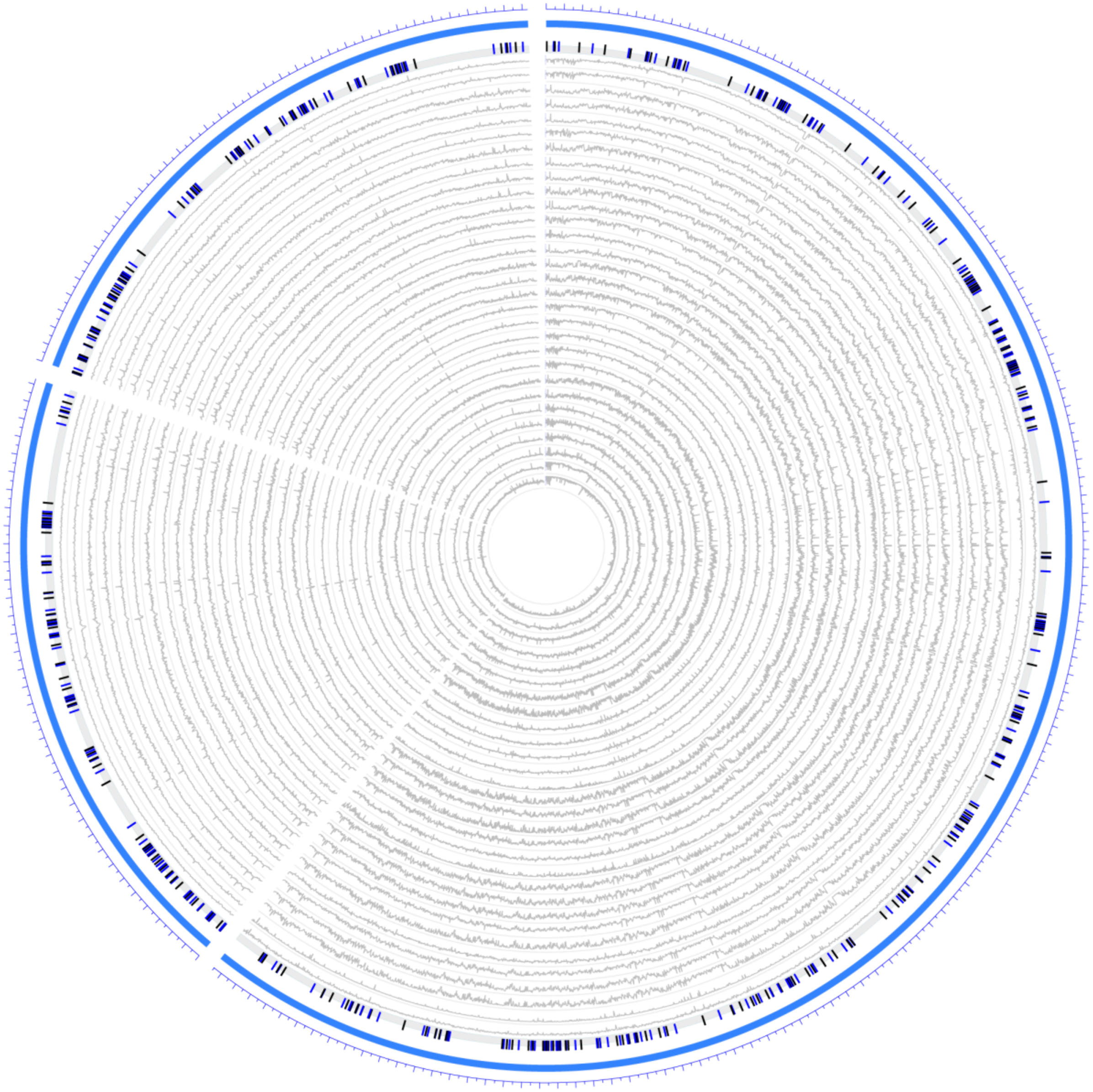

Supplementary Fig. 7. Depth of coverage and gene annotation in kingfishers. Circos plot made with OmicCircos[18] showing first three scaffolds >10 MB in size (scaffolds 60-63, outer blue ring segments) along with locations of annotated genes (black bars, outer track), positively selected genes (M2 model, blue bars), and depth of coverage (averaged over 10-kB windows) for each species mapped to the collared kingfisher reference (gray lines, inner tracks). In each concentric circle (i.e., specimen), depth values range between 0 and 20X, with values >20X clipped for readability. Outer hash marks are drawn every 200-kbp.

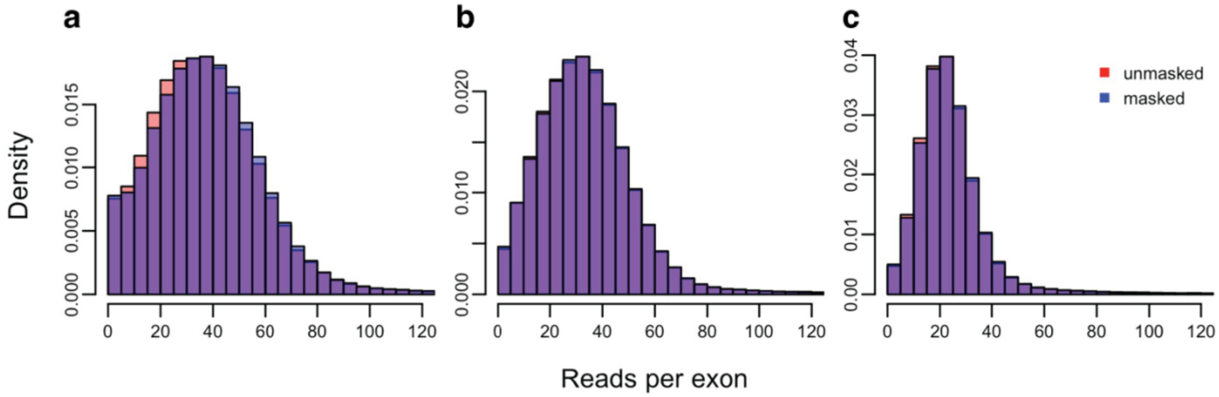

Supplementary Fig. 8. Testing for mapping bias in kingfishers. Results of SNP masking to assess reference read mapping bias. **a-c** Histograms show distribution of reads per exon for the common kingfisher (**a**), the spotted wood kingfisher (**b**), and the pied kingfisher (**c**). These taxa were chosen as exemplars of each of the three major kingfisher clades (see Fig. 1). Large differences in the distribution of per-exon read coverage would indicate mapping bias. Note the larger number of low-coverage reads in the common kingfisher (**a**), which is the most divergent species from the reference collared kingfisher.

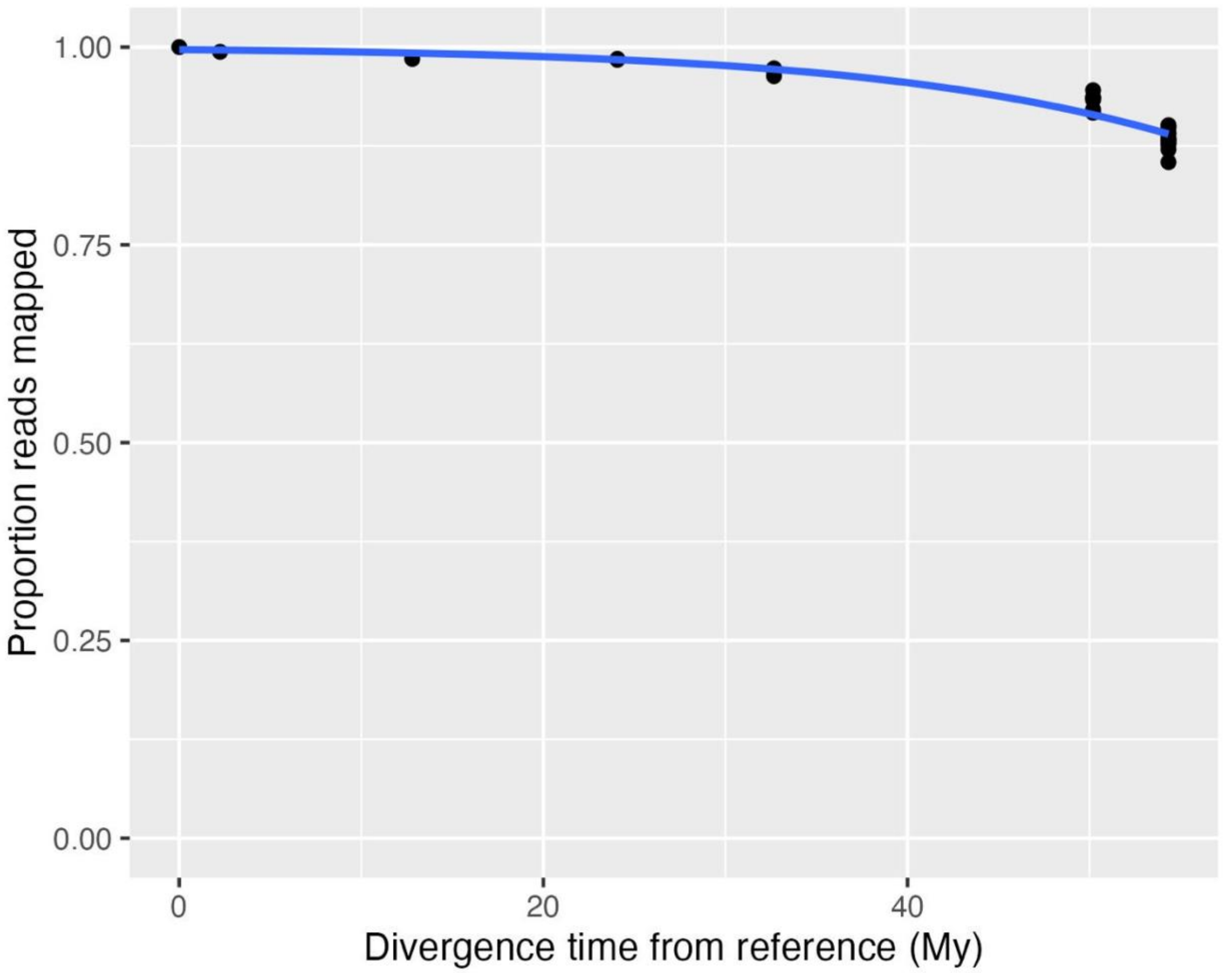

Supplementary Fig. 9. Proportion of kingfisher reads mapped versus divergence time from the collared kingfisher reference genome. Line is a binomial model fit. Values are all above 0.85 (85%) which is comparable with previous benchmarks on read mapping to a reference for divergent species[17]. Note: the outgroup (*Momotus momota*, ~75% reads mapped) was removed from the plot since positive selection results were focused on ingroup kingfishers.

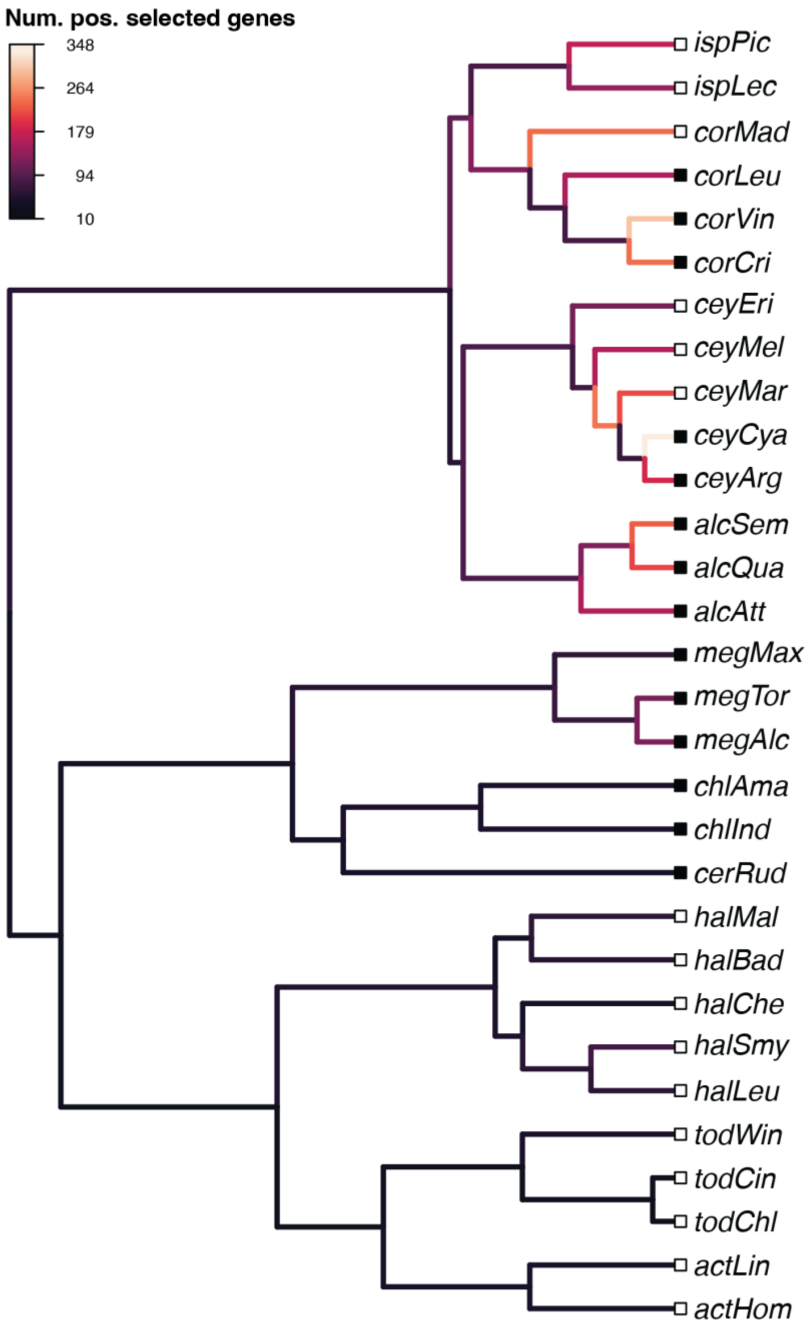

Supplementary Fig. 10. Positive selection in branches of the kingfisher tree. Branch colors indicate number of significant genes showing positive selection in a subset of amino acid sites. See legend for numbers. Rectangles at tips indicate whether species plunge-dive (black) or not (white).

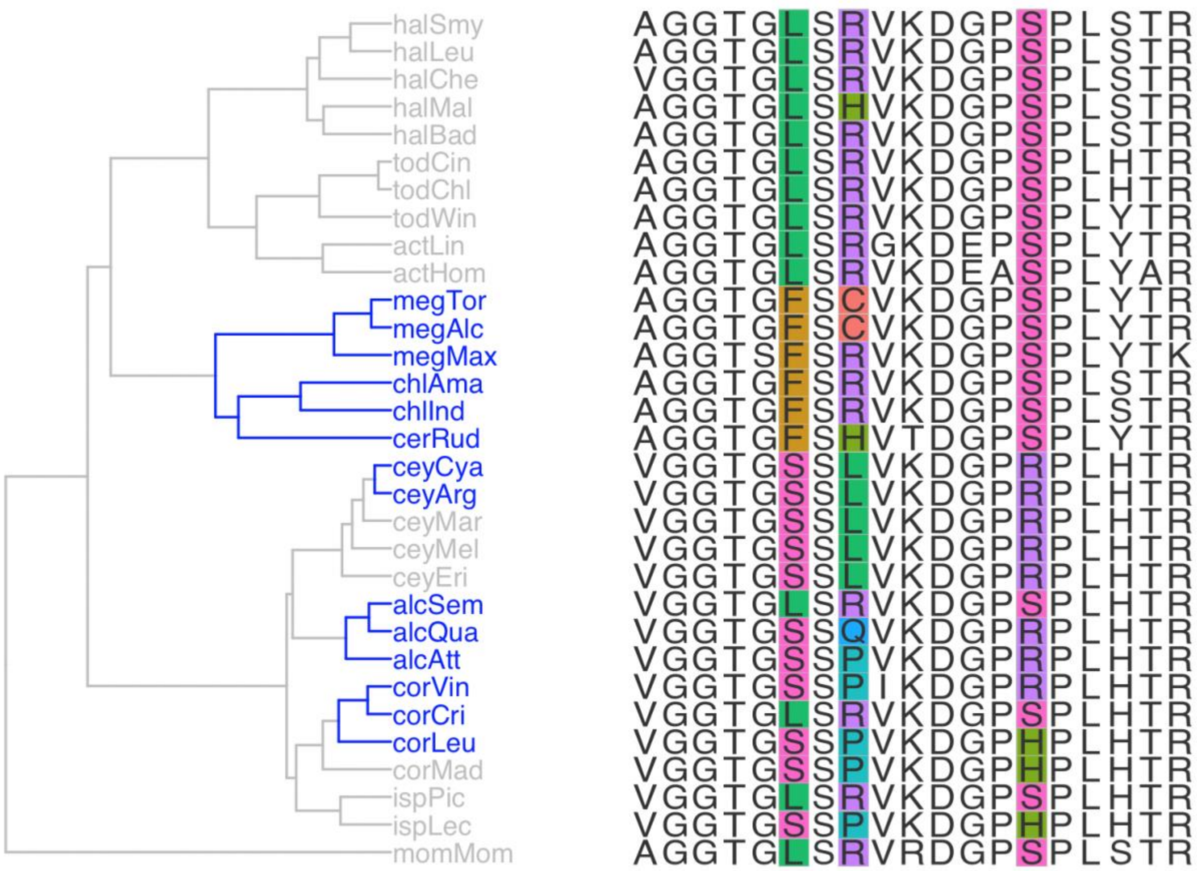

Supplementary Fig. 11. Convergent evolution of the *MAPT* gene. Plot shows the *MAPT* amino acid sequence with positively selected sites (207, 209, 215) highlighted. Branches are colored by whether they plunge-dive (blue) or not (gray).

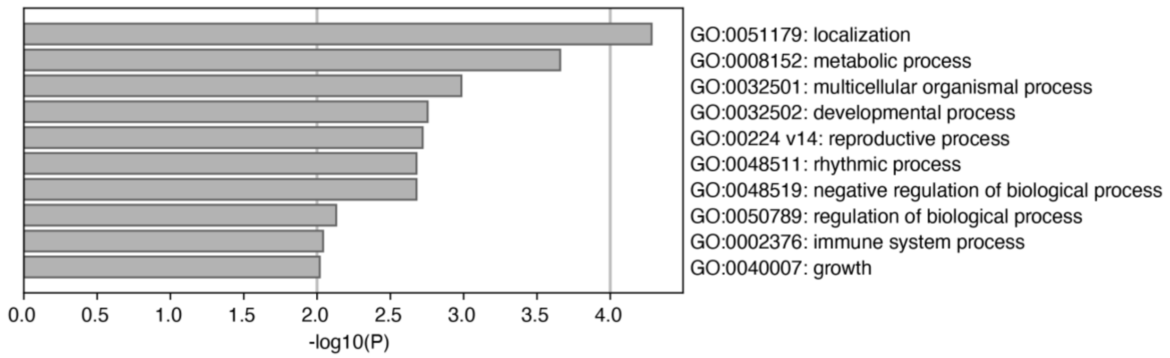

Supplementary Fig. 12. Positive selection scan across kingfishers. Bars show significant levels for enriched gene functions (labels at right) determined as the set of genes that shows positive selection across kingfishers but not convergent within plunge-diving species.

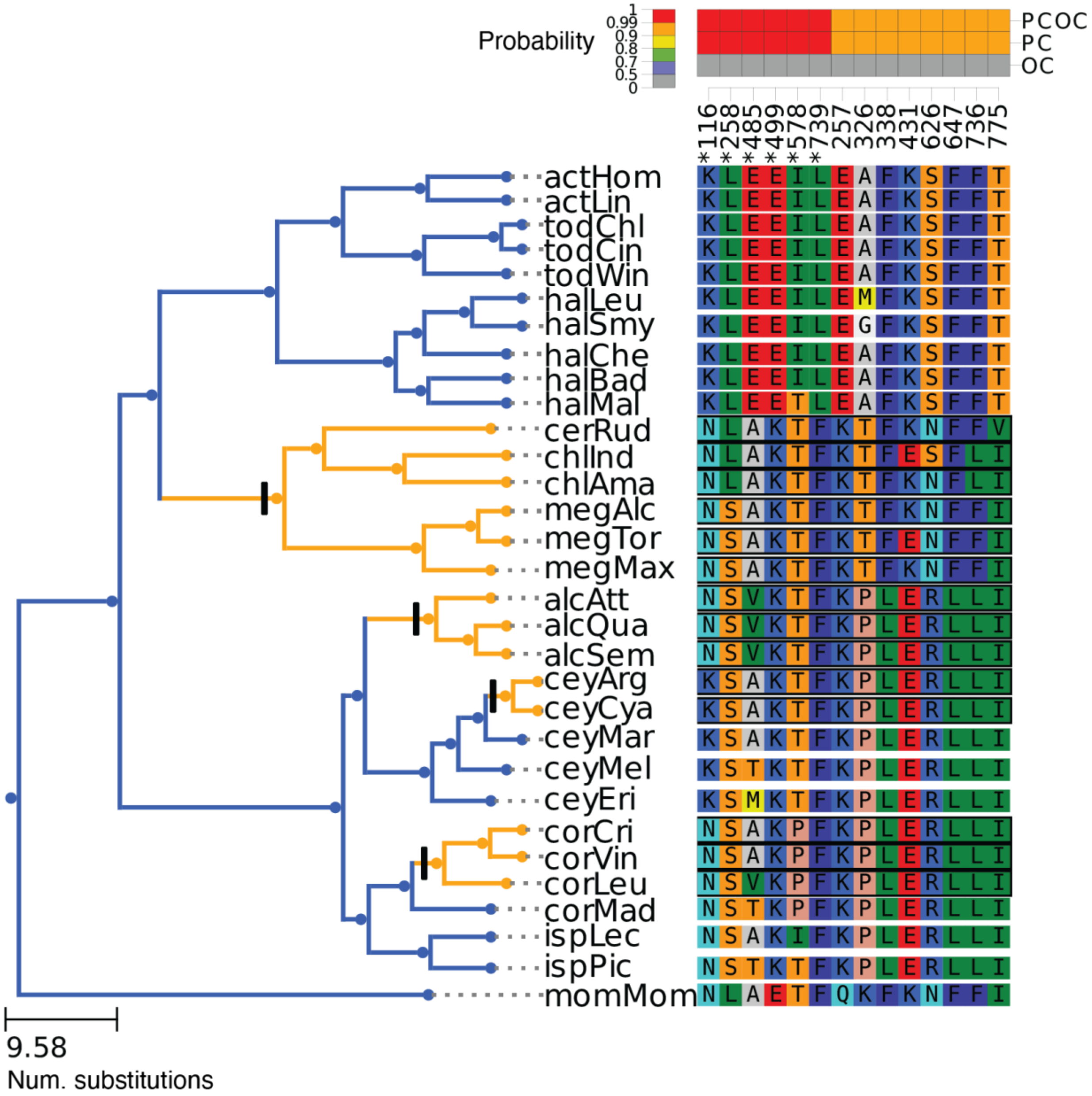

Supplementary Fig. 13. Convergent evolution of umami taste perception in plunge-diving kingfishers. The umami taste receptor gene (*TAS1R3*) shows convergent amino acid sequences at six sites. Other taste receptors did not show this pattern. Lineages are colored by whether they plunge-dive (yellow) or not (blue).

Supplementary Table 1. Museum samples used for genome resequencing. See Supplementary Fig. 7 for depth of coverage details.

Scientific name	Common name	SRR ID	FMNH ID	Coverage	% mapped
Actenoides hombroni	Hombron's kingfisher	10355467	472665	14.6	98.4
Actenoides lindsayi	Spotted wood kingfisher	10366801	449743	18.4	98.5
Alcedo atthis	Common kingfisher	10368067	358313	18.4	90.0
Alcedo quadribrachys	Shining-blue kingfisher	10364708	486866	16.1	88.3
Alcedo semitorquata	Half-collared kingfisher	10368102	455370	12.2	89.0
Ceryle rudis	Pied kingfisher	10360398	455382	15.8	94.6
Ceyx argentatus	Silvery kingfisher	9614116	472663	20.2	90.1
Ceyx cyanopectus	Indigo-banded kingfisher	10352303	474761	11.0	88.2
Ceyx erithaca	Oriental dwarf kingfisher	10377492	484039	14.7	87.1
Ceyx margarethae	Dimorphic dwarf kingfisher	9642614	344953	17.8	88.5
Ceyx melanurus	Philippine dwarf kingfisher	10360281	472748	14.0	87.7
Chloroceryle amazona	Amazon kingfisher	10377295	433227	18.4	93.7
Chloroceryle inda	Green-and-rufous kingfisher	10359626	433229	13.4	93.3
Corythornis cristatus	Malachite kingfisher	10359915	396437	12.2	88.4
Corythornis leucogaster	White-bellied kingfisher	10357240	490150	17.2	89.2
Corythornis madagascariensis	Madagascan pygmy kingfisher	9644342	438655	15.7	85.5
Corythornis vintsioides	Malagasy kingfisher	9644504	345674	19.3	89.7
Halcyon badia	Chocolate-backed kingfisher	10357491	396327	18.0	97.3
Halcyon chelicuti	Striped kingfisher	10352149	447269	14.4	97.0
Halcyon leucocephala	Gray-headed kingfisher	10352182	396443	13.6	96.3
Halcyon malimbica	Blue-breasted kingfisher	10352151	429406	15.6	97.2
Halcyon smyrnensis	White-throated kingfisher	10351892	484041	15.5	97.3
Ispidina lecontei	African dwarf kingfisher	10364840	501554	15.7	87.9
Ispidina picta	African pygmy kingfisher	10352169	474745	12.8	88.0
Megaceryle alcyon	Belted kingfisher	10352524	489166	11.4	92.1
Megaceryle maxima	Giant kingfisher	10352523	486865	14.4	91.7
Megaceryle torquata	Ringed kingfisher	10353979	320982	13.4	93.6
Momotus momota	Amazonian motmot	10354213	473950	10.7	77.3
Todiramphus chloris collaris	Collared kingfisher	21604398	358326	–	–
Todiramphus cinnamominus	Guam kingfisher	10349430	391013	17.0	99.4
Todiramphus winchelli	Winchell's kingfisher	10350663	358321	20.4	98.5

Supplementary Table 2. Positive selection results for the umami gene *TAS1R3*. Comparing different models of protein evolution in PAML using the expanded dataset, including kingfishers and other avian species[5]. Most amino acid sites under positive selection fell outside the transmembrane region, with the exception of amino acid site 700.

Model	lnL	k	AIC	ΔAIC	Sites
M0	-34379	122	69002	2023	–
M1	-33514	3	67034	55	–
M2	-33552	5	67114	135	–
**MA**	**-33485**	**5**	**66979**	**0**	**125, 353, 394, 405, 545, 578, 700, 715**
MA_{null}	-33506	4	67019	40	–
PCOC	–	–	–	–	116, 258, 485, 499, 578
Baldwin et al.[6]	–	–	–	–	206, 237, 372, 387, 511, 530

Supplementary References

1. Pertea, G. & Pertea, M. GFF Utilities: GffRead and GffCompare. *F1000Res.* **9**, 304 (2020).
2. Abzhanov, A. *et al.* The calmodulin pathway and evolution of elongated beak morphology in Darwin's finches. *Nature* **442**, 563–567 (2006).
3. Eliason, C. M., McCullough, J. M., Andersen, M. J. & Hackett, S. J. Accelerated Brain Shape Evolution Is Associated with Rapid Diversification in an Avian Radiation. *Am. Nat.* **197**, 576–591 (2021).
4. Zhao, H., Li, J. & Zhang, J. Molecular evidence for the loss of three basic tastes in penguins. *Curr. Biol.* **25**, R141–R142 (2015).
5. Shultz, A. J. & Sackton, T. B. Immune genes are hotspots of shared positive selection across birds and mammals. *Elife* **8**, (2019).
6. Baldwin, M. W. *et al.* Evolution of sweet taste perception in hummingbirds by transformation of the ancestral umami receptor. *Science* **345**, 929–933 (2014).
7. Lahti, D. C. *et al.* Relaxed selection in the wild. *Trends Ecol. Evol.* **24**, 487–496 (2009).
8. Natarajan, C. *et al.* Predictable convergence in hemoglobin function has unpredictable molecular underpinnings. *Science* **354**, 336–339 (2016).
9. Baldwin, M. W. & Ko, M.-C. Functional evolution of vertebrate sensory receptors. *Horm. Behav.* **124**, 104771 (2020).
10. Tong, C., Avilés, L., Rayor, L. S., Mikheyev, A. S. & Linksvayer, T. A. Genomic signatures of recent convergent transitions to social life in spiders. *Nat. Commun.* **13**, 6967 (2022).
11. Roscito, J. G. *et al.* Convergent and lineage-specific genomic differences in limb regulatory elements in limbless reptile lineages. *Cell Rep.* **38**, 110280 (2022).
12. Sackton, T. B. *et al.* Convergent regulatory evolution and loss of flight in paleognathous birds. *Science* **364**, 74–78 (2019).
13. Cheng, Y. *et al.* Comparative Genomics Reveals Evolution of a Beak Morphology Locus in a High-Altitude Songbird. *Mol. Biol. Evol.* **37**, 2983–2988 (2020).

14. Yusuf, L. *et al.* Noncoding regions underpin avian bill shape diversification at macroevolutionary scales. *Genome Res.* **30**, 553–565 (04/2020).
15. Sackton, T. B. & Clark, N. Convergent evolution in the genomics era: new insights and directions. *Philos. Trans. R. Soc. Lond. B Biol. Sci.* **374**, 20190102 (2019).
16. Mapleson, D., Garcia Accinelli, G., Kettleborough, G., Wright, J. & Clavijo, B. J. KAT: a K-mer analysis toolkit to quality control NGS datasets and genome assemblies. *Bioinformatics* **33**, 574–576 (2017).
17. Sedlazeck, F. J., Rescheneder, P. & von Haeseler, A. NextGenMap: fast and accurate read mapping in highly polymorphic genomes. *Bioinformatics* **29**, 2790–2791 (2013).
18. Hu, Y. *et al.* OmicCircos: A simple-to-use R package for the circular visualization of multidimensional omics data. *Cancer Inform.* **13**, 13–20 (2014).
