# Supplementary material for: Genomic signatures of convergent shifts to plunge-diving behavior in birds
Source: Commun Biol. 2023 Oct 24;6:1011. doi: 10.1038/s42003-023-05359-z (PMC10598022; doi:10.1038/s42003-023-05359-z)
Supplement: Supplementary file 3 — Description of Additional Supplementary Files [file 42003_2023_5359_MOESM3_ESM.pdf]

Description of Additional Supplementary Files

File name: Supplementary Data 1
Description: Gene enrichment results for combined approach.

File name: Supplementary Data 2
Description: Gene enrichment results for aBSREL positive selection method.

File name: Supplementary Data 3
Description: Gene enrichment results for PAML M2 model.

File name: Supplementary Data 4
Description: Gene enrichment results for the drop-test approach for identifying converge positive selection.

File name: Supplementary Data 5
Description: Gene enrichment results for CSUBST analyses of convergent positive selection.
